# ‘I want to be the sort of owner that he wants me to be’: Rationales for biosecurity implementation among British horse owners

**DOI:** 10.1111/evj.14047

**Published:** 2024-01-04

**Authors:** Kelsey L. Spence, Sarah M. Rosanowski, Josh Slater, Jacqueline M. Cardwell

**Affiliations:** ^1^ Veterinary Epidemiology, Economics and Public Health Group Royal Veterinary College Hertfordshire UK; ^2^ Digital Agriculture Grasslands Research Centre, AgResearch Limited Palmerston North New Zealand; ^3^ Melbourne Veterinary School, Faculty of Science University of Melbourne Werribee Victoria Australia

**Keywords:** biosecurity, equine infectious diseases, horse, qualitative research

## Abstract

**Background:**

Horse owners play a critical role in mitigating the risk of pathogen spread between horses. However, little is known about how they view biosecurity and whether they experience barriers to the uptake of preventive measures.

**Objectives:**

To explore horse owners' attitudes, perceptions, and experiences of biosecurity and identify how these factors shape horse owners' decisions for biosecurity implementation.

**Study design:**

Qualitative study using semi‐structured interviews.

**Methods:**

Interviews were conducted with 23 horse owners across Great Britain. Participants were purposively selected to include those in different geographic regions, with different management arrangements, and varied length of horse ownership experience. Interviews were audio‐recorded, transcribed verbatim, and analysed using a critical realist thematic analysis.

**Results:**

Participants felt a moral obligation to prioritise their horse's happiness, which became a challenge when certain biosecurity measures (e.g., quarantine) were perceived as compromising their horse's happiness or comfort (Theme 1). A lack of biosecurity was the social norm among shared yards and competition venues (Theme 2), which made it difficult for participants to implement biosecurity measures effectively on their own. Combined with the sense of moral obligation participants felt towards their horse, this meant that participants had to ‘care double’ (i.e., be more vigilant than they would otherwise) to compensate for collective inaction (Theme 3).

**Main limitations:**

Participants may have been more interested in and/or knowledgeable about biosecurity than the general horse owning population.

**Conclusions:**

The findings highlight several challenges that could be addressed to improve biosecurity implementation among horse owners. Efforts to encourage improved uptake of biosecurity measures should focus on communicating how reducing the risk of disease aligns with horse care. Further research on social norms in the horse industry is needed, in addition to identifying strategies to encourage a collective adoption of biosecurity measures.

## 1 INTRODUCTION

Equine infectious diseases pose significant health, welfare, and economic risks to the British horse industry. Recent outbreaks of equine infectious diseases in the United Kingdom, including equine influenza and equine viral arteritis in 2019, have demonstrated the risks of pathogen spread among horses when participating in activities such as breeding and attending equestrian sport competitions.^1^, ^2^ Other contagious diseases, such as *Streptococcus equi* (strangles), are also commonly reported among horse facilities in the United Kingdom.^3^ The continued occurrence of equine disease outbreaks can have negative consequences for horse owners and facilities, including the closure of equestrian competition venues, loss of revenue, and for some diseases, a high morbidity or mortality rate among affected horses.^4^, ^5^

Biosecurity refers to a set of measures used to prevent or reduce the spread of pathogens.^6^ Biosecurity measures such as isolation, vaccination, cleaning, and disinfection are key to reducing the spread of pathogens that can compromise equine health and welfare.^7^ However, the implementation of biosecurity measures is highly variable across horse owners and horse facilities. Compared with the UK Thoroughbred racing population, which is supported by voluntary codes of practice for biosecurity use,^8^ the non‐racing population is less regulated and may experience a higher variability in uptake of biosecurity measures. Previous studies have reported that even when horse owners are aware of behaviours that constitute good biosecurity, they do not necessarily implement them fully on their premises.^2^, ^9^ Furthermore, some horse owners perceive that contagious diseases pose a low risk to their horse and may therefore be unlikely to engage in disease prevention behaviours.^10^, ^11^, ^12^ This suggests that horse owners may experience barriers to the implementation of biosecurity measures, leading to an increased risk of pathogen spread among horses in the population. However, little is known about which barriers currently exist and how horse owners perceive those barriers, leaving a significant knowledge gap.

Horse owners' implementation of biosecurity depends on a variety of factors, including demographics, situational awareness, perceived risk, previously held beliefs, and sociocultural norms.^10^, ^13^, ^14^, ^15^ These findings are consistent with those from studies of livestock farmers, where psychological factors such as responsibility, experience, risk aversion, and biosecurity knowledge, were associated with good biosecurity behaviours.^16^, ^17^, ^18^ However, further in‐depth analysis is required to identify how these factors might influence the uptake of biosecurity measures among British horse owners. Additionally, further research into how horse owners make decisions about biosecurity in their day‐to‐day contexts is warranted. Therefore, the objectives of this study were to explore horse owners' attitudes, perceptions, and experiences of biosecurity and identify how these factors shape horse owners' decisions to implement biosecurity measures.

## 2 MATERIALS AND METHODS

### 2.1 Theoretical perspective

The study was underpinned by a critical realist ontology and contextualist epistemology. Critical realism asserts that an objective reality exists, but that experiences of reality can differ between people and their contexts.^19^, ^20^ This aligns with a contextualist epistemology, which acknowledges the context‐contingent nature of participant's accounts of reality and, in this case, how that influences their interpretation of biosecurity.^20^, ^21^

### 2.2 Participant selection

Horse owners were eligible to participate if they were 18 years of age or older, lived in the United Kingdom at the time of the study, owned or were primarily responsible for the care of at least one horse, and used their horse(s) for leisure or competition purposes (other than racing). Potential participants were initially identified via a questionnaire of horse owners' perceptions of exotic disease risk.^11^ From those questionnaire respondents who consented to be contacted for follow up by the research team (*n* = 179), potential interviewees were purposively selected to include perspectives across several demographic characteristics, including geographical location, yard type (e.g., shared or sole ownership), and discipline (e.g., leisure or competitive equestrian sport). This was achieved by purposively sampling individuals from each level within each of the three categories. Additional participants were identified through snowball sampling (participant referral of other participants), when convenient and initiated by the participant, to capture views of horse owners who did not previously complete the questionnaire. A total of 56 horse owners were contacted, of which 23 agreed to participate, four declined to participate, and 29 did not respond to the invitation. The research team contacted all potential participants via email to describe the study and invite them to participate in an in‐person interview. Participants were not provided with incentives for participating, but they were offered coffee, tea, and snacks during the interview for hospitality purposes.

### 2.3 Interviews

Semi‐structured interviews were conducted between July and November 2019 by the first author (KLS). The interviewer was a female postdoctoral researcher with expertise in equine biosecurity and training in qualitative methods. The interviewer's background as an epidemiologist and researcher (but neither a horse owner nor a veterinarian) was disclosed to participants prior to the start of the interview. All interviews were conducted face‐to‐face at a location of the participant's choosing; this was most often at their home, a coffee shop, or the location where their horse was kept. Other individuals were often nearby when the interviews took place in public, but only the interviewer and participant were actively engaged in the conversation. Prior to the start of the interview, the interviewer discussed the consent form with the participant and obtained written informed consent to participate. A semi‐structured interview guide, previously piloted with two horse owners to ensure question comprehension, was used to encourage conversation about biosecurity (File S1). The interview guide included open‐ended questions relating to three broad topics: perceptions, attitudes, and decisions relating to biosecurity; experiences of equine infectious diseases; and perspectives on preparing for future disease outbreaks. Repeat interviews were not conducted (i.e., each participant was interviewed only once). Immediately following the interview, the interviewer made notes detailing the context surrounding the interview and any initial insights or observations.

### 2.4 Analysis

Interviews were audio recorded and transcribed intelligent verbatim (i.e., word‐for‐word transcription with light editing to remove pauses and filler words) by an external transcription company. Transcripts were checked for accuracy against the audio recordings by the first author and were not returned to participants for feedback. A critical realist approach to thematic analysis, based on the method described by Fryer,^22^ was conducted by the first author. This method aims to describe causal explanations of phenomena, and therefore aligned with our overall aim of understanding rationales for the uptake of biosecurity measures. First, the transcripts were read to gain familiarity with the data, and notes were made detailing initial observations, thoughts, and questions. Inductive line‐by‐line coding was then used to label topics, concepts, or ideas expressed by participants throughout the transcript. All data and their initial codes were imported into NVivo 12 (Lumivero, LLC) to facilitate data management. Codes were further refined through an iterative process of reflection on the semantic or latent meaning in the data extracts captured by initial codes, followed by standardisation and consolidation of similar codes. Themes were developed through a similarly inductive, iterative, and reflexive process of examining relationships between codes of similar meaning, supported by discussions among the research team. The final themes reflected patterns identified across the dataset. Direct quotes from the interviews are presented in italics, or in quotation marks within the text, to exemplify the constructed themes.

## 3 RESULTS

Interviews were conducted with the final sample of 23 horse owners (Table 1). Interviews were a median of 58 min in length (range, 28–88 min). Three themes were developed from the interview data: (1) A moral obligation to prioritise the horse's happiness; (2) A lack of biosecurity is the social norm; and (3) Needing to ‘care double’ to compensate for collective inaction.

**TABLE 1:** Characteristics of horse owners who participated in qualitative semi‐structured interviews on equine biosecurity.

Owner ID	Gender	Country	Region	Discipline	Yard type
1	Female	England	East	Competition	Livery
2	Female	England	Southwest	Leisure	Private (shared)
3	Female	England	Southwest	Leisure	Livery
4	Female	England	East	Leisure	Private (sole use)
5	Female	England	East	Leisure	Private (shared)
6	Male	England	Northwest	Leisure	Private (sole use)
7	Female	England	Northwest	Competition	Private (sole use)
8	Female	Wales	South	Leisure	Private (shared)
9	Female	England	Southeast	Competition	Livery
10	Female	England	East	Competition	Livery
11	Female	England	East Midlands	Competition	Private (sole use)
12	Female	England	East Midlands	Leisure	Livery
13	Female	England	East	Leisure	Livery
14	Female	Wales	Southeast	Leisure	Private (sole use)
15	Female	England	Northeast	Leisure	Private (shared)
16	Female	England	Northeast	Leisure	Private (sole use)
17	Female	England	Northeast	Leisure	Livery
18	Female	England	Northeast	Competition	Livery
19	Female	England	Northeast	Leisure	Livery
20	Female	England	West Midlands	Competition	Private (sole use)
21	Female	England	Northeast	Leisure	Livery
22	Female	Scotland	East	Competition	Private (sole use)
23	Female	Scotland	Northeast	Competition	Private (sole use)

### 3.1 Theme 1: A moral obligation to prioritise the horse's happiness

Participants regarded themselves as being morally responsible for the care of their horse, and because of that responsibility, decisions surrounding implementing biosecurity measures had to take the horse's happiness or comfort into consideration. Some participants argued that having an ‘ownership’ status came with a duty of care towards their horse and, as such, they had to treat them in the ‘right’ way:

This is a live animal, and we take them on, and in taking them on we take on the responsibility to look after them. They're not wild animals, they're domesticated—she knows when it's time to come in and ‘That's my stable and I'm happy in there’ because that's all she's known. It is our responsibility to look after them and if we're going to buy them, ride them and borrow that freedom, it's our responsibility to do what's right for them.

(Owner 18)

Implementing biosecurity was not always the clear choice for maintaining a horse's happiness or comfort. Biosecurity measures that relied on separation, such as quarantine, isolation, or preventing nose‐to‐nose contact, were sometimes perceived as putting unnecessary restrictions on the horse's ability to ‘be a horse’:

I would never stop him from speaking to another horse because I think that's all part of being a horse. They need to socialise and sniff and that sort of thing.

(Owner 4)

Separation measures were also particularly difficult for the owner to implement if their horse showed visible signs of distress. In these situations, putting them back in the herd was the best option to minimise distress, especially when the perceived risk of pathogen spread was lower than the actual risk of their horse becoming ‘upset’:

When I brought [Horse #1] home, [Horse #2] was here and I had the other two ponies, and I think it was one of the ponies that had the most awful nasal discharge […] We just isolated him, but then he got upset because he was isolated, so we thought, what the hell, put them back together.

(Owner 22)

However, when owners perceived a high risk to their horse, implementing additional biosecurity measures was seen as justifiable to protect the horse's health and minimise potential effects of a disease, even if it meant compromising the horse's happiness. This was particularly true for suspected cases of strangles (*S. equi*), since it was perceived as being especially harmful for horses:

We hadn't been here very long when we thought one of the horses might have strangles. Boy, did this farm kick into action, I'll tell you! I mean, the horse was shut in and nobody was allowed off the farm until they'd dipped their boots and everything. We did all of the things. Anyway, it wasn't strangles, as it happens.

(Owner 17)

In contrast to biosecurity measures that relied on separation, vaccines were generally regarded by participants as an acceptable choice to protect their horse's health while minimising the horse's risk of discomfort. Some participants felt that the effort, cost, and emotional considerations of vaccinating their horse were less than those associated with their horse contracting a disease:

I want to protect my horse and I think an inoculation is cheaper than dealing with [a disease] and getting a vet out every so many days with a very sick pony, and just for the emotional trauma of it, I'd rather pay whatever the inoculation is […] it's a lot cheaper than an emergency call out on a Sunday or a Saturday or a Friday night at 8pm because your horse can't breathe.

(Owner 4)

While participants acknowledged the benefits of vaccines, many questioned the frequency with which they were recommended by veterinarians and equestrian organisations. In 2019, the occurrence of several equine influenza outbreaks prompted a change in the recommended vaccine interval from annually to six monthly. Some participants regarded this change as unnecessary and expressed hesitancy about following the advice because of the perceived potential negative effects of the vaccine on their horse:

There are a lot of anecdotal comments in the barefoot community that a lot of horses are showing up with issues following vaccines. […] I haven't found any of mine directly affected to a large degree. Some degree of footiness, perhaps. And is that an acceptable trade‐off from risking one having tetanus? I think it is. But if we were talking about [vaccinating] them every six months, I think I would question whether I personally would want to do them six monthly knowing that there are background issues which do come up.

(Owner 3)

Identifying risks and benefits of vaccines was challenging for participants, and many expressed difficulties in understanding the nuances surrounding vaccines. This was further complicated by the communications about equine influenza distributed by veterinarians, equestrian bodies, and government agencies, which some horse owners found confusing and lacking sufficient explanation about why vaccines were needed:

People think they do a vaccination and they're not going to get the disease. Then we got to the stage where we understood they might get it, but it might be less serious, and that was fine. But then you had horses that weren't displaying symptoms, they were swabbed because they were going to an event, and lo and behold, they had flu. And they've been vaccinated, so what's the point? You're back to square one.

(Owner 15)

Overall, participants' motivations for choosing whether to implement biosecurity practices tended to prioritise what they perceived the horse wanted and what they felt they owed to their horse:

Horses are a privilege, not a right, so I think we should learn as much as we possibly can and try to keep them, to the best of our ability, the way they're supposed to be kept. […] I want to be the sort of owner that he wants me to be, that's all it is.

(Owner 19)

### 3.2 Theme 2: A lack of biosecurity is the social norm

Many yards had a culture where a lack of routine biosecurity measures was the social norm. A low level, or complete absence, of biosecurity was expected, which made it difficult for individual owners to implement biosecurity measures effectively and on their own. One participant described a situation where a horse was not isolated upon arrival, demonstrating the implicit shared standards of complacency:

There was no isolation prior to a new horse arriving at the yard […] which quite shocked me, actually. I think other people were aware and thought, ‘Oh well, if [isolation] wasn't going to be followed then it wasn't going to be followed.’ I didn't hear anybody try and do anything about it, but then, neither did I […] I assumed, probably quite wrongly, that everybody's horses that were already there were fine, and I knew mine were fine, so I just made the assumption.

(Owner 2)

Many participants found it challenging to confront others about their lack of adherence to existing biosecurity measures or guidelines, or had unsuccessfully attempted to address situations where others on the yard did not want to observe good biosecurity:

When we had strangles, they put a disinfectant mat across where the infected horses were for the girls to walk on. But because they muck out in [sports shoes], they would step over the mats so they didn't get their feet wet. It's really hard to police things. The yard manager should have yelled at them and said, ‘That's there for a reason, wipe your feet’. But it was just people like me looking and thinking, ‘Oh God’, and trying to make a joke saying, ‘You just stepped over that’, and then they just say, ‘Yeah, but I've got a hole in my shoe’, and you think, ‘Okay’.

(Owner 1)

Because of the difficulties in challenging the social norms around biosecurity, participants felt that these issues had to be addressed indirectly, for example, by modelling behaviours with other horse owners, rather than confrontationally:

If you go in and immediately start telling people that leaving dirty sponges and things kicking around all over the yard is a really stupid thing to do, then obviously that gets people's backs up. But if you can go in and you quietly tidy up and you're seen to be sweeping up, and you make sure that the bowls are scrubbed when people have fed their horses, and you perhaps offer to scrub other people's bowls that are sharing the same storage spaces with you, then at least you're doing your little bit to feel that you're reducing the potential transmission of any problems, and it might make other people think about their hygiene and just up their game a little bit too.

(Owner 3)

Participants observed a similar attitude towards biosecurity at equestrian competitions. While many venues had requirements for engaging in biosecurity measures (e.g., vaccination certificates and passports), these were often not enforced by competition organisers. Participants expressed frustration with the lack of accountability and the apparent assumption that people would implement biosecurity on their own:

I think there's just an assumption that people will adhere to the guidelines. Actually, my vaccinations have just come out of date. They're a month late. I don't think a month late is an issue and my vet's going to come out and do them all, but nobody knows that. […] I have thought about saying, ‘When are we going to get checked, is anyone going to check [at the competition]?’ But all [the organisers] do is send emails out saying they need to be up to date and you could be checked. But that's not enough, really.

(Owner 7)

Social norms also existed around discussing cases of disease among the community. If an outbreak occurred, there was a desire to keep it quiet while it was being managed. Some horse owners felt judged and stigmatised by other owners when their horse did get a disease, especially if they went against the norm of keeping it quiet. This was compounded by the lack of official reporting of non‐notifiable diseases (e.g., strangles), as horse owners would speculate about outbreaks on social media:

People were saying to me ‘There is no strangles in this area’ and I said, ‘Well there is, because it's my horse’ […] It really knocks your confidence when you've got people who don't know you, they're judging you on Facebook and social media […] It was awful because no matter what I said, there was somebody with something negative to say. But I think you have to do it, I think you just have to have one whistleblower that's willing to stand up and say … If we don't stand up and say we've got it, then it spreads.

(Owner 19)

Recognising that diseases were part of owning horses, and could happen just by chance, was perceived as helpful to challenge the stigma associated with disease:

I think people are still quite ashamed of [strangles]. And you go, well, these things just happen. They're unfortunate. It's like I've learned with colic, I always thought colic was because you're a bad horse owner, you've not looked after your horse correctly. Now that my horse has had three bouts of colic, I know that's not the case. Or I'm really that bad [laughing]. I don't think I'm that bad. So, I think it's the same with strangles.

(Owner 9)

### 3.3 Theme 3: Needing to ‘care double’ to compensate for collective inaction

Some participants expressed a willingness to implement better or additional biosecurity practices but felt it would be challenging to do so without the agreement of all horse owners at the yard. Biosecurity was perceived as something everyone had to participate in to be effective, and as such, the spread and control of pathogens depended on the collective actions (or inactions) of those within the community:

I don't actually believe in lots of yard rules, but I might do other things like infection control and vaccinations and things of that ilk, because that protects everybody. I may not want to do it but by doing it I protect your horse. I might be willing to take the risk but why should I put your horse at risk because I'm not willing to take reasonable health steps?

(Owner 6)

Participants were aware that they could not control other horse owners' actions, but still worried about how others' actions impacted the level of risk posed to their own horses. Since infectious diseases could be transmitted among horses, stopping the spread depended on population‐level action. Those who kept their horse on their own private yard described how this lessened their worries about others' inactions, since they could implement whichever biosecurity measures they deemed appropriate:

I always felt that one of the reasons I really wanted my own land was because I could keep him how I wanted to keep him. When you keep them at livery, you don't get to choose what horses they're in with, you don't get to choose the practices of who's worming when.

(Owner 16)

In contrast, participants who kept their horse at shared yards (such as livery yards) had to consider the involvement of other horse owners in either managing, or contributing to, the care of their horse. Participants' own actions were not always sufficient to protect their horse, and having someone else in charge of their horse's management often led to a sense of vulnerability:

I didn't realise how hard it was having two animals that I love so much somewhere that isn't mine. And there's been times where I've felt … if you're upset about something, you can't do anything. I feel like you're almost vulnerable because you are at someone's mercy with the creatures that you love, and you don't want anything to happen to them.

(Owner 8)

There was also a perception that some horse owners were unwilling to change their behaviour, even if they were aware it put other people's horses at risk. Some suggested this might be due to selfishness or being ‘set in their ways’:

One of the vets very kindly gave up his Saturday morning to come [to the yard], and anybody who wanted to could go and have a little mini meet and greet and a chat about biosecurity, and quite a few of the owners went, but some were very notable by their absence. And those were the ones that you know would never change. No matter what was said or done, they would never change their habits. […] They weren't uneducated people, they were just, I think, very set in their ways and very selfish. They didn't really have any care for other people or other people's horses or anything else.

(Owner 3)

Furthermore, information shared by other horse owners was also seen to put participants' horses at risk. While it was thought that some misinformation originated from novice owners who lacked basic horse welfare knowledge, long‐time horse owners who refused to change their habits were also suspected of contributing to it. In either case, identifying whether biosecurity information was rooted in truth or tradition presented a significant challenge:

Who do you trust, who do you know is telling the truth? It's like old wives' tales, it's like Robin Hood, people think it, then it becomes the truth, and it's not. Over the years it's been twisted and turned and other things added to it and it just becomes this ball of bollocks, basically. And people are like, ‘But it's always been like that’. They don't understand that it doesn't always have to be like that.

(Owner 12)

Whether because of ignorance, selfishness, or entrenched habit, if other horse owners did not take measures to protect their own horse, it seemed highly unlikely they would implement measures to protect the health of other people's horses:

If they don't care about their own animals, I don't suppose they care about someone else's, but yeah, for me that's the thing, I feel like I do everything for my horses, but sometimes it's … what about all the people that don't? How do we protect our horses from them?

(Owner 15)

The inactions of some other horse owners were therefore perceived as a direct threat, which motivated participants to implement additional measures to keep their horse(s) safe:

People don't care about other people, and because they don't care about what they're doing, I have to care double about my own horse. That's an awful judgment on society, but I think it is a bit like that.

(Owner 8)

## 4 DISCUSSION

This study provides key insights into the motivations for, and challenges to, the adoption of biosecurity measures among horse owners. The findings suggest that horse owners are highly motivated to provide care for their horse in a way that minimises their horse's discomfort and maximises their happiness, which sometimes conflicts with the implementation of biosecurity measures. A horse owner's moral responsibility for their horse motivates them to implement biosecurity measures in certain situations, especially if threats to their horse's health are perceived. However, some perceived threats included the inaction of other horse owners and a socially accepted lack of biosecurity among the wider horse industry, which caused difficulties for participants to implement biosecurity measures effectively on their own. These findings indicate that the decision to implement biosecurity measures is not straightforward, which may cause challenges for disease preparedness within the horse industry.

Participants tended to prioritise the happiness of their horse over implementing certain biosecurity measures, especially if the measure was viewed as restrictive (e.g., quarantine) or the situation was perceived as low risk (e.g., absence of an ongoing outbreak). While there is no consensus on what constitutes ‘happiness’ among animals, it can be inferred from their affective states, where a ‘happy’ animal is one which expresses a positive affective state most of the time.^23^ This definition aligns with a ‘Quality of Life’ framework of animal welfare, which ensures an animal has a ‘life worth living’.^24^ It is important to note that study participants focused on descriptions of their horse's happiness rather than their horse's overall welfare, which suggest that animal welfare frameworks that prioritise both physical and affective states may be better adopted by horse owners. A study of UK leisure horses found that owners tried to balance their horse's physical and mental health when assessing wellbeing.^25^ These findings are consistent with the Five Domains Model of animal welfare, which outlines how subjective experiences, specifically positive experiences that enhance quality of life, contribute to an animal's overall welfare.^24^ A study of working equids found that those whose owners believed horses felt emotions were in significantly better health than those whose owners did not have this belief,^26^ suggesting that attribution of ‘happiness’ to horses may in fact improve their welfare. However, horse owners' ability to infer happiness from their horse's demeanour may be inconsistent. A previous study of horse owners reported that while the vast majority of participants said they could tell when their horse was happy, many participants misinterpreted signs of distress as signs of happiness.^27^ Further research on horse owners' interpretation of horse happiness, and how this interpretation impacts their perception of overall horse welfare, is required.

A main motivating factor behind horse care decisions was a moral obligation held by participants to provide their horse with optimal care according to what they perceived their horse wanted. These findings align with a previous study of British horse owners which reported that horse owners felt moral and ethical obligations towards their horses and that this influenced their purchasing and preventive health care decisions.^28^ Theories of the human–animal bond have suggested that companion animal owners feel ‘duties of guardianship’ which includes moral responsibilities to, and for, their pets.^29^ Humans feel strong companionship bonds with their horses, which subsequently motivates them to protect their horse from external threats.^30^, ^31^ A positive human–animal bond has also been found to influence management practices in other industries. A study of pig farmers found that pig welfare and productivity was associated with farmers' beliefs of the importance of the pig on their farm, subsequently leading to better husbandry practices.^32^ This suggests that communication strategies that emphasise the role biosecurity plays in protecting a horse's health and maintaining the human–horse bond may be beneficial to improve uptake among owners.

An alternative approach to communicating how biosecurity aligns with horse care may also be beneficial. A previous study reported that horse owners associated the use of preventive measures with being a ‘responsible’ horse owner,^10^ which has been echoed in other livestock industries where the uptake of biosecurity measures was considered as part of a ‘good farmer’ identity.^33^, ^34^ However, our findings suggest that some horse owners view this responsibility as an obligation to do what they perceive their horse wants, which may or may not include implementing biosecurity measures. Exploring how responsibility is conceptualised among owners, including how the horse's perceived desires are taken into consideration, may help to better understand horse owners' motivations for horse care. Furthermore, a shift in how biosecurity among horse owners is conceptualised or communicated may be needed. For example, a study exploring biosecurity responsibilities among livestock farmers described how farmers underwent a ‘choreography of care’, which consisted of routine care practices and rituals that were responsive to their animals.^35^ These care practices, such as regularly monitoring herd health and administering vaccines, were consistent with biosecurity measures even though farmers did not conceptualise them as such.^35^ Given the hesitation of some participants to apply biosecurity measures during low‐risk situations, communicating how biosecurity measures align with care practices that benefit the horse may be useful. Additionally, a review of current biosecurity practices may be needed to identify which measures align with protecting horses from disease while also minimising emotional discomfort.

Social norms, which are the social patterns that govern behaviour, were found to be highly influential in establishing the acceptable use of biosecurity. A general lack of biosecurity among British horse owners has previously been reported, demonstrating a consistent social norm of expected behaviour across the industry.^7^, ^10^, ^12^, ^36^, ^37^ Research during the COVID‐19 pandemic found that livery yards had deeply embedded practices of care for their horses, which were ingrained ways of doing things that were considered difficult to change even when required.^38^ Because multiple owners can keep their horses at a single shared yard, they must make judgements about how to care for their horse within a wider sociocultural context.^39^ This explains why it might be difficult to challenge embedded social norms on yards, especially if horse owners want to avoid judgement for not conforming to these norms.^39^ The tendency to conform to social norms depends on cultural ‘tightness’ or ‘looseness’, which refers to the degree to which cultures form social norms and tolerate deviances from these norms.^40^ Research on adherence to mask‐wearing during the COVID‐19 pandemic found that tight cultures (i.e., those with higher social conformity) were more likely to initially adopt mask‐wearing and continue this behaviour once policies were dropped.^41^ Study participants reported difficulties in challenging the social norm of a lack of biosecurity, suggesting there may be low tolerance for deviations from this norm among shared yards with tight cultures.

Among livestock farmers, the uptake of biosecurity measures has been described as governed by social scripts, where farmers consciously and sub‐consciously follow rules and adopt values and behaviours determined by society.^42^ By adopting these values and behaviours, farmers refine their world views (i.e., what they believe to be true) which further reinforces social norms.^43^ Changes to embedded social norms often begin when an individual stops complying with a norm, which is subsequently aided by other influential individuals who stop enforcing the norm.^44^ Importantly, this change must come from the community itself rather than solely implemented or enforced by authoritative figures (e.g., the government).^44^ Among horse owners, changes to social norms could be motivated by a small group of horse owners and afterwards aided by those in a position of influence (e.g., yard owners/managers and equestrian competition organisers). However, this would likely require collective action among horse owners, which could be a challenge, as described by study participants. Because of the lack of collective action to implement biosecurity, participants felt they needed to put in extra effort to care for their horse, since others' inactions posed risks to their horse. Among cattle and sheep farmers, barriers to collective action during an outbreak were linked to a lack of social cohesion and trust among farmers.^45^ Similarly, prior studies exploring the British horse industry demonstrated a lack of cohesion among its members, which subsequently caused challenges to disease preparedness.^10^, ^12^ Further research on collective action towards biosecurity in the horse industry, and strategies to improve social cohesion, is recommended to determine where changes can be targeted to improve uptake.

When assessing qualitative research, readers should consider the trustworthiness of the findings, including whether they are credible (interpretations make sense in the context described), dependable (methodology is clearly described), confirmable (interpretations are supported by the data presented), and transferable (are likely to be applicable in other contexts).^46^, ^47^ It is important to note that the biosecurity experiences, perceptions, and attitudes reported in this study may differ from those of other groups of horse owners. Qualitative research findings are not statistically generalisable to a target population, but instead provide in‐depth insights from a smaller group of individuals purposively selected to capture a variety of characteristics.^48^ Most of our study participants (all but one participant) identified as female, lived in England, and were likely interested in biosecurity given that they agreed to participate in the study. This should be considered when interpreting the findings, as they may be more transferable to horse owners that share these characteristics. Furthermore, the perspectives of the research team inherently influenced the findings, since qualitative researchers actively engage in data collection, conceptualisation, and interpretation.^49^ While the interviews were conducted in 2019, the research team analysed the data during the COVID‐19 pandemic, which may have influenced how the data were interpreted. However, the consistencies across participants' accounts, and the parallels demonstrated across pre‐pandemic studies of horse owners' and livestock farmers' perspectives of biosecurity, support the reliability of the findings.^49^

## 5 CONCLUSIONS

This study sought to explore horse owners' attitudes, perceptions, and experiences of biosecurity to identify rationales for the uptake of biosecurity measures. The findings suggest that while horse owners are highly motivated to provide optimal care for their horse, biosecurity may not always align with the type of care they perceive their horse wants. The decision to implement biosecurity is further complicated by existing social norms and a lack of collective action. This highlights several barriers to optimal biosecurity. Efforts to communicate the benefits of biosecurity should focus on how reducing the risk of disease aligns with horse care. Further research on social norms in the horse industry, and strategies to encourage a collective change in biosecurity engagement, is required.

## AUTHOR CONTRIBUTIONS

Sarah M. Rosanowski, Josh Slater, and Jacqueline M. Cardwell obtained funding for this research. Kelsey L. Spence, Sarah M. Rosanowski, Josh Slater, and Jacqueline M. Cardwell contributed to study design. Kelsey L. Spence collected and analysed the data, with guidance from Jacqueline M. Cardwell. Kelsey L. Spence, Sarah M. Rosanowski, Josh Slater, and Jacqueline M. Cardwell contributed to data interpretation and manuscript preparation. All authors approved the final manuscript.

## FUNDING INFORMATION

Alborada Trust.

## CONFLICT OF INTEREST STATEMENT

The authors declare no conflicts of interest.

## DATA INTEGRITY STATEMENT

Kelsey L. Spence had full access to all data and takes responsibility for the integrity of the data and accuracy of the data analysis.

## ETHICAL ANIMAL RESEARCH

This study was granted institutional ethical approval from the Royal Veterinary College (URN SR2019‐0227).

## INFORMED CONSENT

All participants provided written informed consent to participate.

### PEER REVIEW

The peer review history for this article is available at https://www.webofscience.com/api/gateway/wos/peer-review/10.1111/evj.14047.

## Supporting information


**File S1.** Interview guide.

## Data Availability

The data that support the findings of this study are not publicly available due to privacy or ethical restrictions.
