# Supplementary material for: ‘I want to be the sort of owner that he wants me to be’: Rationales for biosecurity implementation among British horse owners
Source: Equine Vet J. 2024 Jan 4;57(1):183–92. doi: 10.1111/evj.14047 (PMC11616952; doi:10.1111/evj.14047)
Supplement: Supplementary file 1 — File S1. Interview guide. [file EVJ-57-183-s001.pdf]

File S1:

INTERVIEW GUIDE – EXPLORE PHASE 2

Introduction

- Tell me about how you first got involved with horses.

Attitudes towards biosecurity

- When you hear the word ‘biosecurity’, what does it mean to you?
- What sorts of things do you currently do to avoid disease?
    - Is there anything that you’re not currently doing, but would like to do?
    - Is there anything that you feel you should be doing?
- How do you decide which measures you’re going to implement?

Experiences of infectious diseases

- Have you had any experiences with infectious diseases in the past?
- What are you most concerned about when you think about infectious diseases on your yard / in your local area / in the UK?
- Which infectious diseases are you most concerned about, and why?

Preparing for exotic diseases

- What comes to mind when you think about exotic diseases?
    - What mental images do you have of exotic diseases?
    - Have you heard of exotic diseases before? In what context?
- How do you think exotic diseases compare with the diseases you normally deal with?
- What do you expect the response to be to an exotic disease outbreak in the UK from other owners / yards / government?
- What could be done to prepare for an exotic disease outbreak?
